# A review of deep learning applications for genomic selection

**DOI:** 10.1186/s12864-020-07319-x

**Published:** 2021-01-06

**Authors:** Osval Antonio Montesinos-López, Abelardo Montesinos-López, Paulino Pérez-Rodríguez, José Alberto Barrón-López, Johannes W. R. Martini, Silvia Berenice Fajardo-Flores, Laura S. Gaytan-Lugo, Pedro C. Santana-Mancilla, José Crossa

**Affiliations:** 1 Facultad de Telemática, Universidad de Colima, 28040 Colima, Colima Mexico; 2 Departamento de Matemáticas, Centro Universitario de Ciencias Exactas e Ingenierías (CUCEI), Universidad de Guadalajara, 44430 Guadalajara, Jalisco Mexico; 3 Colegio de Postgraduados, CP 56230 Montecillos, Edo. de México Mexico; 4 Department of Animal Production (DPA), Universidad Nacional Agraria La Molina, Av. La Molina s/n La Molina, 15024 Lima, Peru; 5 Biometrics and Statistics Unit, International Maize and Wheat Improvement Center (CIMMYT), Km 45, CP 52640 Carretera Mexico-Veracruz, Mexico; 6 School of Mechanical and Electrical Engineering, Universidad de Colima, 28040 Colima, Colima Mexico

**Keywords:** Genomic selection, Deep learning, Plant breeding, Genomic trends

## Abstract

**Background:**

Several conventional genomic Bayesian (or no Bayesian) prediction methods have been proposed including the standard additive genetic effect model for which the variance components are estimated with mixed model equations. In recent years, deep learning (DL) methods have been considered in the context of genomic prediction. The DL methods are nonparametric models providing flexibility to adapt to complicated associations between data and output with the ability to adapt to very complex patterns.

**Main body:**

We review the applications of deep learning (DL) methods in genomic selection (GS) to obtain a meta-picture of GS performance and highlight how these tools can help solve challenging plant breeding problems. We also provide general guidance for the effective use of DL methods including the fundamentals of DL and the requirements for its appropriate use. We discuss the pros and cons of this technique compared to traditional genomic prediction approaches as well as the current trends in DL applications.

**Conclusions:**

The main requirement for using DL is the quality and sufficiently large training data. Although, based on current literature GS in plant and animal breeding we did not find clear superiority of DL in terms of prediction power compared to conventional genome based prediction models. Nevertheless, there are clear evidences that DL algorithms capture nonlinear patterns more efficiently than conventional genome based. Deep learning algorithms are able to integrate data from different sources as is usually needed in GS assisted breeding and it shows the ability for improving prediction accuracy for large plant breeding data. It is important to apply DL to large training-testing data sets.

## Background

Plant breeding is a key component of strategies aimed at securing a stable food supply for the growing human population, which is projected to reach 9.5 billion people by 2050 [1, 2]. To be able to keep pace with the expected increase in food demand in the coming years, plant breeding has to deliver the highest rates of genetic gain to maximize its contribution to increasing agricultural productivity. In this context, an essential step is harnessing the potential of novel methodologies. Today, genomic selection (GS), proposed by Bernardo [3] and Meuwissen et al. [4] has become an established methodology in breeding. The underlying concept is based on the use of genome-wide DNA variation (“markers”) together with phenotypic information from an observed population to predict the phenotypic values of an unobserved population. With the decrease in genotyping costs, GS has become a standard tool in many plant and animal breeding programs with the main application of reducing the length of breeding cycles [5–9].

Many empirical studies have shown that GS can increase the selection gain per year when used appropriately. For example, Vivek et al. [10] compared GS to conventional phenotypic selection (PS) for maize, and found that the gain per cycle under drought conditions was 0.27 (t/ha) when using PS, which increased to 0.50 (t/ha) when GS was implemented. Divided by the cycle length, the genetic gain per year under drought conditions was 0.067 (PS) compared to 0.124 (GS). Analogously, under optimal conditions, the gain increased from 0.34 (PS) to 0.55 (GS) per cycle, which translates to 0.084 (PS) and 0.140 (GS) per year. Also for maize, Môro et al. [11] reported a similar selection gain when using GS or PS. For soybean [*Glycine max* (L.) Merr.], Smallwood et al. [12] found that GS outperformed PS for fatty acid traits, whereas no significant differences were found for traits yield, protein and oil. In barley, Salam and Smith [13] reported similar (per cycle) selection gains when using GS or PS, but with the advantage that GS shortened the breeding cycle and lowered the costs. GS has also been used for breeding forest tree species such as eucalyptus, pine, and poplar [14]. Breeding research at the International Maize and Wheat Improvement Center (CIMMYT) has shown that GS can reduce the breeding cycle by at least half and produce lines with significantly increased agronomic performance [15]. Moreover, GS has been implemented in breeding programs for legume crops such as pea, chickpea, groundnut, and pigeon pea [16]. Other studies have considered the use of GS for strawberry [17], cassava [18], soybean [19], cacao [20], barley [21], millet [22], carrot [23], banana [24], maize [25], wheat [26], rice [27] and sugar cane [28].

Although genomic best linear unbiased prediction (GBLUP) is in practice the most popular method that is often equated with genomic prediction, genomic prediction can be based on any method that can capture the association between the genotypic data and associated phenotypes (or breeding values) of a training set. By fitting the association, the statistical model “learns” how the genotypic information maps to the quantity that we would like to predict. Consequently, many genomic prediction methods have been proposed. According to Van Vleck [29], the standard additive genetic effect model is the aforementioned GBLUP for which the variance components have to be estimated and the mixed model equations of Henderson [30] have to be solved. Alternatively, Bayesian methods with different priors using Markov Chain Monte Carlo methods to determine required parameters are very popular [31–33]. In recent years, different types of (deep) learning methods have been considered for their performance in the context of genomic prediction. DL is a type of machine learning (ML) approach that is a subfield of artificial intelligence (AI). The main difference between DL methods and conventional statistical learning methods is that DL methods are nonparametric models providing tremendous flexibility to adapt to complicated associations between data and output. A particular strength is the ability to adapt to hidden patterns of unknown structure that therefore could not be incorporated into a parametric model at the beginning [34].

There is plenty of empirical evidence of the power of DL as a tool for developing AI systems, products, devices, apps, etc. These products are found anywhere from social sciences to natural sciences, including technological applications in agriculture, finance, medicine, computer vision, and natural language processing. Many “high technology” products, such as autonomous cars, robots, chatbots, devices for text-to-speech conversion [35, 36], speech recognition systems, digital assistants [37] or the strategy of artificial challengers in digital versions of chess, Jeopardy, GO and poker [38], are based on DL. In addition, there are medical applications for identifying and classifying cancer or dermatology problems, among others. For instance, Menden et al. [39] applied a DL method to predict the viability of a cancer cell line exposed to a drug. Alipanahi et al. [40] used DL with a convolutional network architecture to predict specificities of DNA- and RNA-binding proteins. Tavanaei et al. [41] used a DL method for predicting tumor suppressor genes and oncogenes. DL methods have also made accurate predictions of single-cell DNA methylation states [42]. In the genomic domain, most of the applications concern functional genomics, such as predicting the sequence specificity of DNA- and RNA-binding proteins, methylation status, gene expression, and control of splicing [43]. DL has been especially successful when applied to regulatory genomics, by using architectures directly adapted from modern computer vision and natural language processing applications. There are also successful applications of DL for high-throughput plant phenotyping [44]; a complete review of these applications is provided by Jiang and Li [44].

Due to the ever-increasing volume of data in plant breeding and to the power of DL applications in many other domains of science, DL techniques have also been evaluated in terms of prediction performance in GS. Often the results are mixed below the –perhaps exaggerated– expectations for datasets with relatively small numbers of individuals [45]. Here we review DL applications for GS to provide a meta-picture of their potential in terms of prediction performance compared to conventional genomic prediction models. We include an introduction to DL fundamentals and its requirements in terms of data size, tuning process, knowledge, type of input, computational resources, etc., to apply DL successfully. We also analyze the pros and cons of this technique compared to conventional genomic prediction models, as well as future trends using this technique.

## Main body

### The fundamentals of deep learning models

**Fig. 1:**
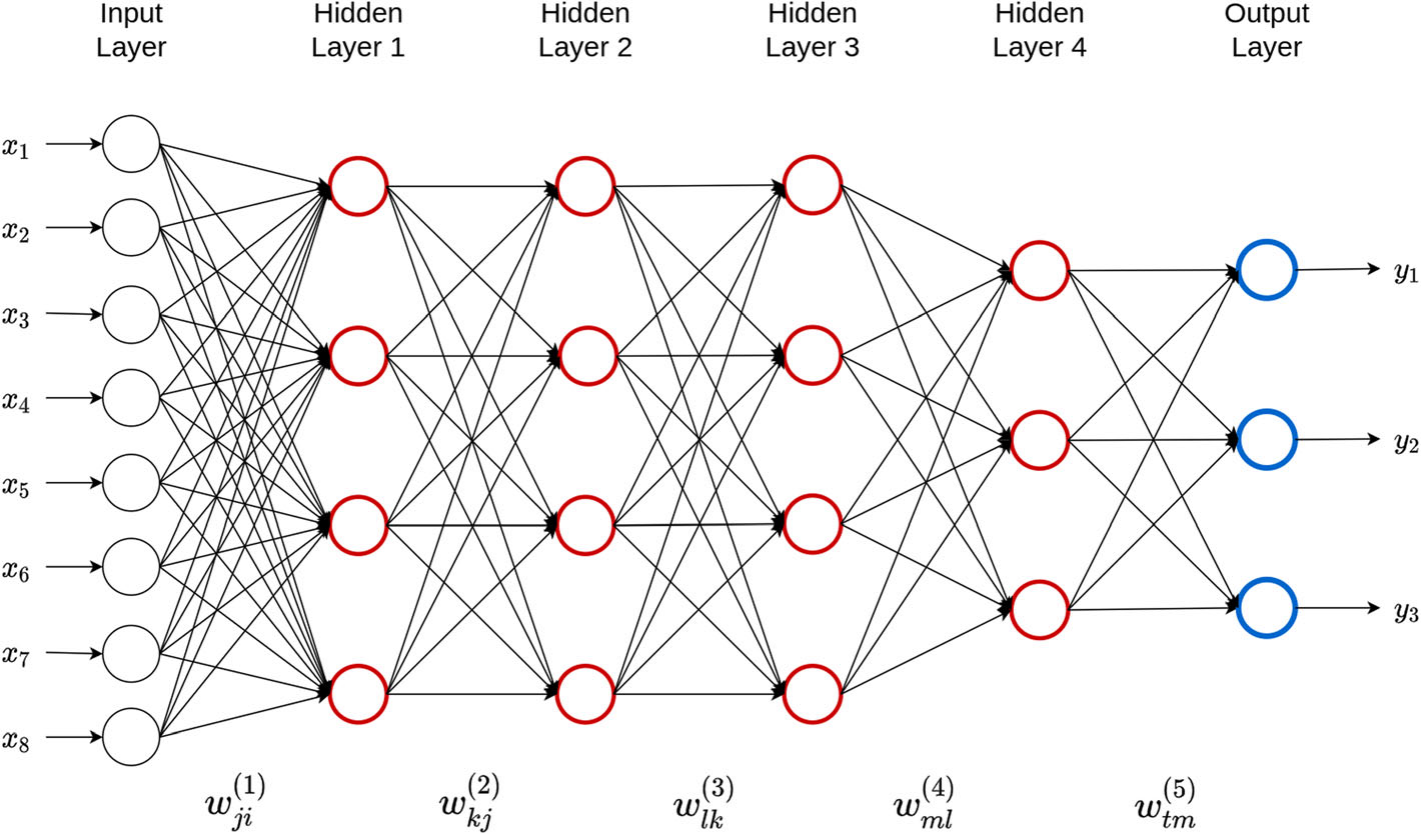
A five-layer feedforward deep neural network with one input layer, four hidden layers and one output layer. There are eight neurons in the input layer that corresponds to the input information, four neurons in the first three hidden layers, three neurons in the fourth hidden layer and three neurons in the output layer that corresponds to the traits that will be predicted

DL models are subsets of statistical “semi-parametric inference models” and they generalize artificial neural networks by stacking multiple processing hidden layers, each of which is composed of many neurons (see Fig. 1). The adjective “deep” is related to the way knowledge is acquired [36] through successive layers of representations. DL methods are based on multilayer (“deep”) *artificial neural networks* in which different nodes (“neurons”) receive input from the layer of lower hierarchical level which is activated according to set activation rules [35–37] (Fig. 1). The activation again defines the output sent to the next layer, which receives the information as input. The neurons in each layer receive the output of the neurons in the previous layer as input. The strength of a connection is called weight, which is a weighting factor that reflects its importance. If a connection has zero weight, a neuron does not have any influence on the corresponding neuron in the next layer. The impact is excitatory when the weight is positive, or inhibitory when the weight is negative. Thus, deep neural networks (DNN) can be seen as directed graphs whose *nodes* correspond to neurons and whose *edges* correspond to the *links* between them. Each neuron receives, as input, a weighted sum of the outputs of the neurons connected to its incoming edges [46].

The deep neural network provided in Fig. 1 is very popular; it is called a feedforward neural network or multi-layer perceptron (MLP). The topology shown in Fig. 1 contains eight inputs, one output layer and four hidden layers. The input is passed to the neurons in the first hidden layer, and then each hidden neuron produces an output that is used as an input for each of the neurons in the second hidden layer. Similarly, the output of each neuron in the second hidden layer is used as an input for each neuron in the third hidden layer; this process is done in a similar way in the remaining hidden layers. Finally, the output of each neuron in the four hidden layers is used as an input to obtain the predicted values of the three traits of interest. It is important to point out that in each of the hidden layers, we attained a weighted sum of the inputs and weights (including the intercept), which is called the net input, to which a transformation called activation function is applied to produce the output of each hidden neuron.

The analytical formulas of the model given in Fig. 1 for *three* outputs, *d* inputs (not only 8), *N*_1_ hidden neurons (units) in hidden layer 1, *N*_2_ hidden units in hidden layer 2, *N*_3_ hidden units in hidden layer 3, *N*_4_ hidden units in hidden layer 4, and three neurons in the output layers are given by the following eqs. (1–5):

1 $$ {V}_{1j}={f}_1\left(\sum \limits_{i=1}^d{w}_{ji}^{(1)}{x}_i+{b}_{j1}\right)\ \mathrm{for}\ j=1,\dots, {N}_1 $$

2 $$ {V}_{2k}={f}_2\left(\sum \limits_{j=1}^{N_1}{w}_{kj}^{(2)}{V}_{1j}+{b}_{k2}\right)\ \mathrm{for}\ k=1,\dots, {N}_2 $$

3 $$ {V}_{3l}={f}_3\left(\sum \limits_{k=1}^{N_2}{w}_{lk}^{(3)}{V}_{2k}+{b}_{l3}\right)\ \mathrm{for}\ l=1,\dots, {N}_3 $$

4 $$ {V}_{4m}={f}_4\left(\sum \limits_{l=1}^{N_3}{w}_{ml}^{(4)}{V}_{3l}+{b}_{m4}\right)\ \mathrm{for}\ m=1,\dots, {N}_4 $$

5 $$ {y}_t={f}_{5t}\left(\sum \limits_{m=1}^{N_4}{w}_{tm}^{(5)}{V}_{4m}+{b}_{t5}\right)\ \mathrm{for}\ t=1,2,3 $$

where *f*_1_, *f*_2_, *f*_3_, *f*_4_ and *f*_5*t*_ are activation functions for the first, second, third, fourth, and output layers, respectively. Eq. (1) produces the output of each of the neurons in the first hidden layer, eq. (2) produces the output of each of the neurons in the second hidden layer, eq. (3) produces the output of each of the neurons in the third hidden layer, eq. (4) produces the output of each of the neurons in the four hidden layer, and finally, eq. (5) produces the output of the response variables of interest. The learning process involves updating the weights (${w}_{ji}^{(1)},{w}_{kj}^{(2)},{w}_{lk}^{(3)},{w}_{ml}^{(4)},{w}_{tm}^{(5)}\Big)$ and biases (*b*_*j*1_, *b*_*k*2_, *b*_*l*3_, *b*_*m*4_, *b*_*t*5_) to minimize the loss function, and these weights and biases correspond to the first hidden layer (${w}_{ji}^{(1)},{b}_{j1}\Big)$, second hidden layer (${w}_{kj}^{(2)},{b}_{k2}\Big)$, third hidden layer (${w}_{lk}^{(3)},{b}_{l3}\Big)$, fourth hidden layer (${w}_{ml}^{(4)},{b}_{m4}\Big)$, and to the output layer (${w}_{tm}^{(5)},{b}_{t5}\Big)$, respectively. To obtain the outputs of each of the neurons in the four hidden layers (*f*_1_, *f*_2_, *f*_3_, and *f*_4_), we can use the rectified linear activation unit (RELU) or other nonlinear activation functions (sigmoid, hyperbolic tangent, leaky_ReLu, etc.) [47–49]. However, for the output layer, we need to use activation functions (*f*_5*t*_) according to the type of response variable (for example, linear for continuous outcomes, sigmoid for binary outcomes, softmax for categorical outcomes and exponential for count data).

It is important to point out that when only one outcome is present in Fig. 1, this model is reduced to a univariate model, but when there are two or more outcomes, the DL model is multivariate. Also, to better understand the language of deep neural networks, next we define the depth, the size and the width of a DNN. The “depth” of a neural network is defined as the number of layers that it contains, excluding the input layer. For this reason, the “depth” of the network shown in Fig. 1 is 5 (4 hidden layers + 1 output layer). The “size” of the network is defined as the total number of neurons that form the DNN; in this case, it is equal to |9 + 5 + 5 + 5 + 4 + 3| = 31. It is important to point out that in each layer (except the output layer), we added + 1 to the observed neurons to represent the neuron of the bias (or intercept). Finally, we define the “width” of the DNN as the layer that contains the largest number of neurons, which, in this case, is the input layer; for this reason, the width of this DNN is equal to 9. Finally, note that the theoretical support for DL models is given by the universal approximation theorem, which states that a neural network with enough hidden units can approximate any arbitrary functional relationships [50–54].

### Popular DL topologies

The most popular topologies in DL are the aforementioned feedforward network (Fig. 1), recurrent neural networks and convolutional neural networks. Details of each are given next.

#### Feedforward networks (or multilayer perceptrons; MLPs)

In this type of artificial deep neural network, the information flows in a single direction from the input neurons through the processing layers to the output layer. Every neuron of layer *i* is connected only to neurons of layer *i* + 1, and all the connection edges can have different weights. This means that there are no connections between neurons in the same layer (no intralayer), and that there are also no connections that transmit data from a higher layer to a lower layer, that is, no supralayer connections (Fig. 1). This type of artificial deep neural network is the simplest to train; it usually performs well for a variety of applications, and is suitable for generic prediction problems where it is assumed that there is no special relationship among the input information. However, these networks are prone to overfitting. Feedforward networks are also called fully connected networks or MLP.

#### Recurrent neural networks (RNN)

**Fig. 2:**
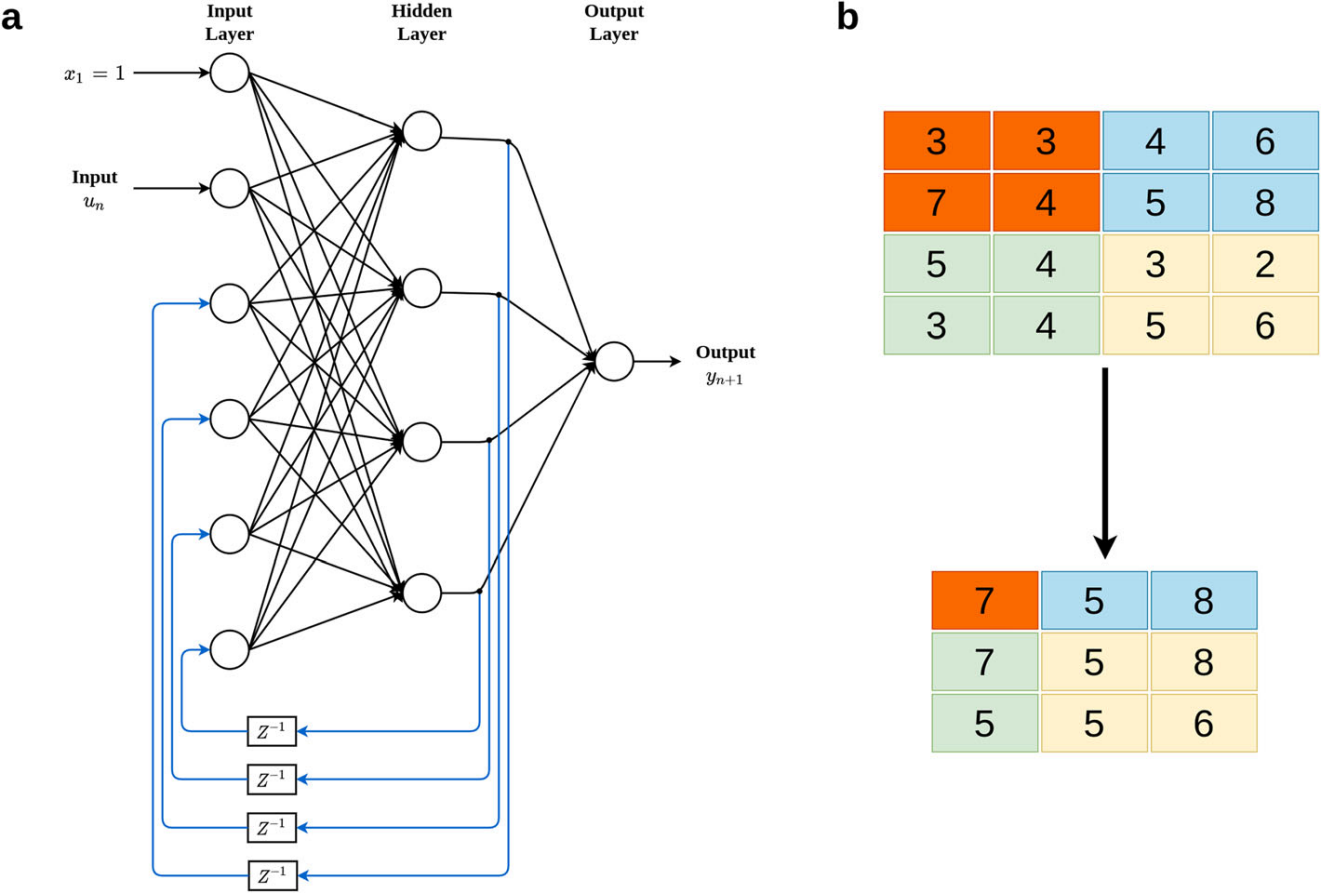
A simple two-layer recurrent artificial neural network with univariate outcome (**a**). Max pooling with 2 × 2 filters and stride 1 (**b**)

In this type of neural network, information does not always flow in one direction, since it can feed back into previous layers through synaptic connections. This type of neural network can be monolayer or multilayer. In this network, all the neurons have: (1) incoming connections emanating from all the neurons in the previous layer, (2) ongoing connections leading to all the neurons in the subsequent layer, and (3) recurrent connections that propagate information between neurons of the same layer. RNN are different from a feedforward neural network in that they have at least one feedback loop because the signals travel in both directions. This type of network is frequently used in time series prediction since short-term memory, or delay, increases the power of recurrent networks immensely, but they require a lot of computational resources when being trained. Figure 2a illustrates an example of a recurrent two-layer neural network. The output of each neuron is passed through a delay unit and then taken to all the neurons, except itself. Here, only one input variable is presented to the input units, the feedforward flow is computed, and the outputs are feedback as auxiliary inputs. This leads to a different set of hidden unit activations, new output activations, and so on. Ultimately, the activations stabilize, and the final output values are used for predictions.

#### Convolutional neural networks (CNN)

CNN are very powerful tools for performing visual recognition tasks because they are very efficient at capturing the spatial and temporal dependencies of the input. CNN use images as input and take advantage of the grid structure of the data. The efficiency of CNN can be attributed in part to the fact that the fitting process reduces the number of parameters that need to be estimated due to the reduction in the size of the input and parameter sharing since the input is connected only to some neurons. Instead of fully connected layers like the feedforward networks explained above (Fig. 1), CNN apply convolutional layers which most of the time involve the following three operations: *convolution, nonlinear transformation and pooling.* Convolution is a type of linear mathematical operation that is performed on two matrices to produce a third one that is usually interpreted as a filtered version of one of the original matrices [48]; the output of this operation is a matrix called feature map. The goal of the pooling operation is to progressively reduce the spatial size of the representation to reduce the amount of parameters and computation in the network. The pooling layer operates on each feature map independently. The pooling operation performs down sampling and the most popular pooling operation is max pooling. The max pooling operation summarizes the input as the maximum within a rectangular neighborhood, but does not introduce any new parameters to the CNN; for this reason, max pooling performs dimensional reduction and de-noising. Figure 2b illustrates how the pooling operation is performed, where we can see that the original matrix of order 4 × 4 is reduced to a dimension of 3 × 3.

**Fig. 3:**
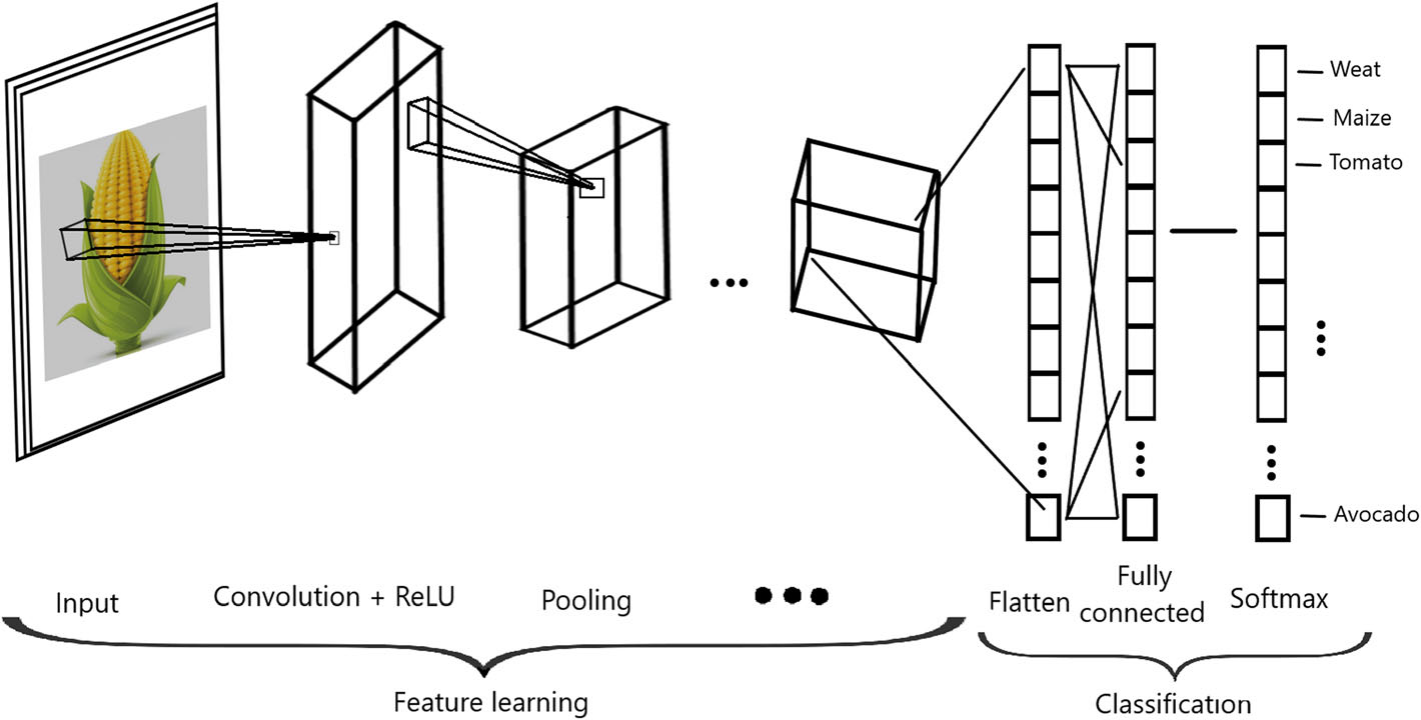
Convolutional neural network

Figure 3 shows the three stages that conform a convolutional layer in more detail. First, the convolution operation is applied to the input, followed by a nonlinear transformation (like Linear, ReLU, hyperbolic tangent, or another activation function); then the pooling operation is applied. With this convolutional layer, we significantly reduce the size of the input without relevant loss of information. The convolutional layer picks up different signals of the image by passing many filters over each image, which is key for reducing the size of the original image (input) without losing critical information, and in early convolutional layers we capture the edges of the image. For this reason, CNN include fewer parameters to be determined in the learning process, that is, at most half of the parameters that are needed by a feedforward deep network (as in Fig. 1). The reduction in parameters has a positive side effect of reducing the training times. Also, Fig. 3 indicates that depending on the complexity of the input (images), the number of convolutional layers can be more than one to be able to capture low-level details with more precision. In Fig. 3 also shows that after the convolutional layers, the input of the image is flattened (flattening layer), and finally, a feedforward deep network is applied to exploit the high-level features learned from input images to predict the response variables of interest (Fig. 3).

### Activation functions

Activation functions are crucial in DL models. Activation functions determine the type of output (continuous, binary, categorical and count) of a DL model and play an important role in capturing nonlinear patterns of the input data. Next, we provide brief details of some commonly used activation functions and suggest when they can be used.

#### Linear

The linear activation function is the identity function. It is defined as *g*(*z*) = *z*, where the dependent variable has a direct, proportional relationship with the independent variable. Thus the output is equal to the input; this activation function is suggested for continuous response variables (outputs) and is used mostly in the output layer [47]. A limitation of this activation function is that it is not capable of capturing nonlinear patterns in the input data; for this reason, it is mostly used in the output layer [47].

#### Rectifier linear unit (ReLU)

The rectifier linear unit (ReLU) activation function is flat below some thresholds and then linear. When the input is below zero, the output is zero, but when the input rises above a certain threshold, it has a linear relationship with the dependent variable *g*(*z*) =  *max* (0, *z*). This activation function is able to capture nonlinear patterns and for this reason, most of the time it is used in hidden layers [47, 48]. This activation function is one of the most popular in DL applications for capturing nonlinear patterns in hidden layers [47, 48]. This activation function has the Dying ReLU problem that occurs when inputs approach zero, or are negative, that causes the gradient of the function becomes zero; thus under these circumstances, the network cannot perform backpropagation and cannot learn efficiently [47, 48].

#### Leaky ReLU

The Leaky ReLU is a variant of ReLU and is defined as $$ g(z)=\left\{\begin{array}{c}z\  ifz>0\\ {}\alpha z\  otherwise\end{array}\right. $$. As opposed to having the function be zero when *z* < 0, the leaky ReLU instead has a small negative slope, *α*, where alpha (*α*) is a value between 0 and 1. This activation function most of the time is also a good alternative for hidden layers because this activation function attempts to fix the problem by having a small negative slope which is called the “dying ReLU” [47]. Sometimes this activation function provides non-consistent predictions for negative input values [47].

#### Sigmoid

A sigmoid activation function is defined as *g*(*z*) = (1 + *e*^−*z*^)^−1^, and maps independent variables near infinite range into simple probabilities between 0 and 1. This activation function is used to capture nonlinear patterns in hidden layers and produce the outputs in terms of probability; for this reason, it is used in the output layers when the response variable is binary [47, 48]. This activation function is not a good alternative for hidden layers because it produces the vanishing gradient problem that slows the convergence of the DL model [47, 48].

#### Softmax

The softmax activation function defined as $g\left({z}_j\right)=\frac{\exp \left({z}_j\right)}{1+{\sum}_{c=1}^C\exp \left({z}_c\right)}$, *j = 1,..,C*, is a generalization of the sigmoid activation function that handles multinomial labeling system; that is, it is appropriate for categorical outcomes. It also has the property that the sum of the probabilities of all the categories is equal to one. Softmax is the function you will often find in the output layer of a classifier with more than two categories [47, 48]. This activation function is recommended only in the output layer [47, 48].

#### Tanh

The hyperbolic tangent (Tanh) activation function is defined as $\tanh \left(\mathrm{z}\right)=\sinh \left(\mathrm{z}\right)/\cosh \left(\mathrm{z}\right)=\frac{\exp (z)-\exp \left(-z\right)}{\exp (z)+\exp \left(-z\right)}$. Like the sigmoid activation function, the hyperbolic tangent has a sigmoidal (“S” shaped) output, with the advantage that it is less likely to get “stuck” than the sigmoid activation function since its output values are between − 1 and 1. For this reason, this activation function is recommended for hidden layers and output layers for predicting response variables in the interval between − 1 and 1 [47, 48]. The vanishing gradient problem is sometimes present in this activation function, but it is less common and problematic than when the sigmoid activation function is used in hidden layers [47, 48].

#### Exponential

This activation function handles count outcomes because it guarantees positive outcomes. Exponential is the function often used in the output layer for the prediction of count data. The exponential activation function is defined as *g*(*z*) =  *exp* (*z*).

### Tuning hyper-parameters

**Fig. 4:**
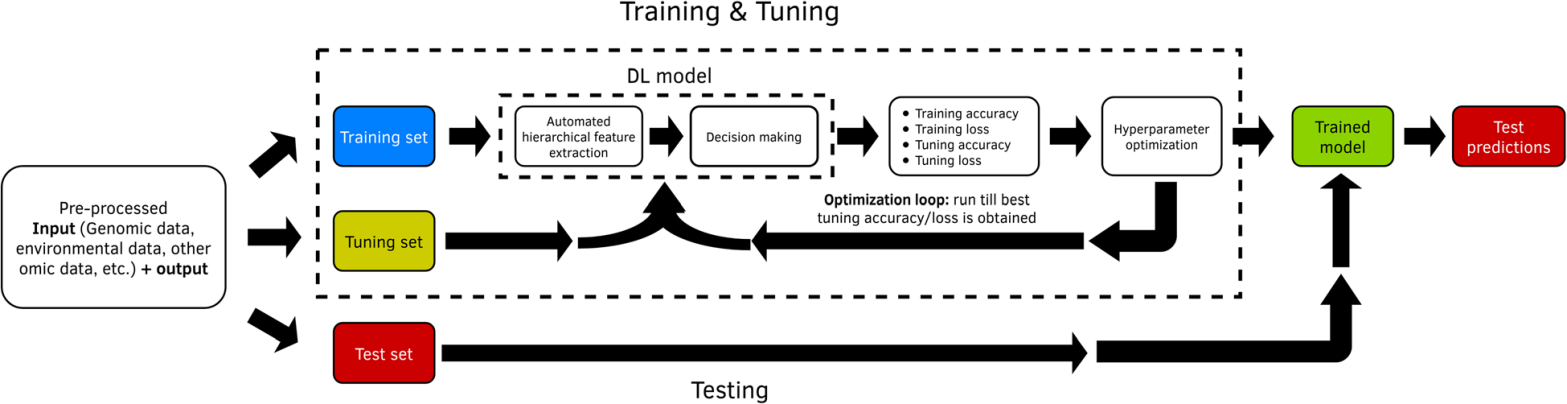
Training set, tuning set and testing set (adapted from Singh et al., 2018)

For training DL models, we need to distinguish between learnable (structure) parameters and non-learnable (hyper-parameters) parameters. Learnable parameters are learned by the DL algorithm during the training process (like weights and bias), while hyper-parameters are set before the user begins the learning process, which means that hyper-parameters (like number of neurons in hidden layers, number of hidden layers, type of activation function, etc.) are not learned by the DL (or machine learning) method. Hyper-parameters govern many aspects of the behavior of DL models, since different hyper-parameters often result in significantly different performance. However, a good choice of hyper-parameters is challenging; for this reason, most of the time a tuning process is required for choosing the hyper-parameter values. The tuning process is a critical and time-consuming aspect of the DL training process and a key element for the quality of the final predictions. Hyper-parameter tuning consists of selecting the optimal hyper-parameter combination from a grid of values with different hyper-parameter combinations. To implement the hyper-parameter tuning process, dividing the data at hand into three mutually exclusive parts (Fig. 4) is recommended [55]:

- a training set (for training the algorithm to learn the learnable parameters),
- a tuning set (for tuning hyper-parameters and selecting the optimal non-learnable parameters), and.
- a testing or validation set (for estimating the generalization performance of the algorithm).

This partition reflects our objective of producing a generalization of the learned structures to unseen data (Fig. 4). When the dataset is large, it can be enough to use only one partition of the dataset at hand (training-tuning-testing). For example, you can use 70% for training, 15% for tuning and the remaining 15% for testing. However, when the dataset is small, this process needs to be replicated, and the average of the predictions in the testing set of all these replications should be reported as the prediction performance. Also, when the dataset is small, and after obtaining the optimal combination of hyper-parameters in each replication, we suggest refitting the model by joining the training set and the tuning set, and then performing the predictions on the testing set with the final fitted model. One approach for building the training-tuning-testing set is to use conventional k fold (or random partition) cross-validation where k-1 folds are used for the training (outer training) and the remaining fold for testing. Then inside each fold with the corresponding training, k-fold cross-validation is used, and k-1 folds are used for training (inner training) and the remaining fold for tuning evaluation. The model for each hyper-parameter combination in the grid is trained with the inner training data set, and the combination in the grid with the lower prediction error is selected as the optimal hyper-parameter in each fold. Then if the sample size is small using the outer training set, the DL model is fitted again with the optimal hyper-parameter. Finally, with these estimated parameters (weights and bias), the predictions for the testing set are obtained. This process is repeated in each fold and the average prediction performance of the k testing set is reported as prediction performance. Also, it is feasible to estimate a kind of nonlinear breeding values, with the estimated parameters, but with the limitation that the estimated parameters in general are not interpretable as in linear regression models.

### DL frameworks

DL with univariate or multivariate outcomes can be implemented in the Keras library as front-end and Tensorflow as back-end [48] in a very user-friendly way. Another popular framework for DL is MXNet, which is efficient and flexible and allows mixing symbolic programming and imperative programming to maximize efficiency and productivity [56]. Efficient DL implementations can also be performed in PyTorch [57] and Chainer [58], but these frameworks are better for advanced implementations. Keras in R or Python are friendly frameworks that can be used by plant breeders for implementing DL; however, although they are considered high-level frameworks, the user still needs to have a basic understanding of the fundamentals of DL models to be able to do successful implementations. Since the user needs to specify the type of activation functions for the layers (hidden and output), the appropriate loss function, and the appropriate metrics to evaluate the validation set, the number of hidden layers needs to be added manually by the user; he/she also has to choose the appropriate set of hyper-parameters for the tuning process.

Thanks to the availability of more frameworks for implementing DL algorithms, the democratization of this tool will continue in the coming years since every day there are more user-friendly and open-source frameworks that, in a more automatic way and with only some lines of code, allow the straightforward implementation of sophisticated DL models in any domain of science. This trend is really nice, since in this way, this powerful tool can be used by any professional without a strong background in computer science or mathematics. Finally, since our goal is not to provide an exhaustive review of DL frameworks, those interested in learning more details about DL frameworks should read [47, 48, 59, 60].

### Publications about DL applied to genomic selection

Table 1 gives some publications of DL in the context of GS. The publications are ordered by year, and for each publication, the Table gives the crop in which DL was applied, the DL topology used, the response variable used and the conventional genomic prediction models with which the DL model was compared. These publications were selected under the inclusion criterion that DL must be applied exclusively to GS.

**Table 1:** DL application to genomic selection

Obs	Year	Authors	Crop	Topology	Response variable(s)	Comparison with
1	2011	Gianola et al. [61]	Wheat and Jersey cows	MLP	Grain yield (GY), fat yield, milk yield, protein yield, fat yield	Bayesian Ridge regression (BRR)
2	2012	Pérez-Rodríguez et al. [62]	Wheat	MLP	GY and days to heading (DTHD)	BL, BayesA, BayesB, BRR, Reproducing Kernel Hilbert Spaces (RKHS) regression
3	2012	Gonzalez-Camacho et al. [6]	Maize	MLP	GY, female flowering (FFL) or days to silking, male flowering time (MFL) or days to anthesis, and anthesis-silking interval (ASI)	RKHS regression, BL
4	2015	Ehret et al. [63]	Holstein-Friesian and German Fleckvih cattle	MLP	Milk yield, protein yield, and fat yield	GBLUP
5	2016	Gonzalez-Camacho et al. [64]	Maize and wheat	MLP	GY	Probabilistic neural network (PNN)
6	2016	McDowell [65]	Arabidopsis, maize and wheat	MLP	Days to flowering, dry matter, grain yield (GY), spike grain, time to young microspore.	OLS, RR, LR, ER, BRR
7	2017	Rachmatia et al. [66]	Maize	DBN	GY, female flowering (FFL) (or days to silking), male flowering (MFL) (or days to anthesis), and the anthesis-silking interval (ASI)	RKHS, BL and GBLUP
8	2018	Ma et al. [67]	Wheat	CNN and MLP	Grain length (GL), grain width (GW), thousand-kernel weight (TW), grain protein (GP), and plant height (PH)	RR-BLUP, GBLUP
9	2018	Waldmann [68]	Pig data and TLMAS2010 data	MLP	Trait number of live born piglets	GBLUP, BL
10	2018	Montesinos-López et al. [70]	Maize and wheat	MLP	Grain yield	GBLUP
11	2018	Montesinos-López et al. [71]	Maize and wheat	MLP	Grain yield (GY), anthesis-silking interval (ASI), PH, days to heading (DTHD), days to maturity (DTMT)	BMTME
12	2018	Bellot et al. [72]	Human traits	MLP and CNN	Height and bone heel mineral density	BayesB, BRR
13	2019	Montesinos-López et al. [73]	Wheat	MLP	GY, DTHD, DTMT, PH, lodging, grain color (GC), leaf rust and stripe rust	SVM, TGBLUP
14	2019	Montesinos-López et al. [74]	Wheat	MLP	GY, DH, PH	GBLUP
15	2019	Khaki and Wang [75]	Maize	MLP	GY, check yield, yield difference	LR, regression tree
16	2019	Azodi et al. [77]	6 species	MLP	18 traits	rrBLUP, BRR, BA, BB, BL, SVM, GTB
17	2019	Liu et al. [78]	Soybean	CNN	GY, protein, oil, moisture, PH	rrBLUP, BRR, BayesA, BL
18	2020	Abdollahi-Arpanahi et al. [79]	Holstein bulls	MLP and CNN	Sire conception rate	GBLUP, BayesB and RF
19	2020	Zingaretti et al. [80]	Strawberry and blueberry	MLP and CNN	Average fruit weight, early marketable yield, total marketable weight, soluble solid content, percentage of culled fruit	RKHS, BRR, BL,
22	2020	Montesinos-López et al. [81]	Wheat	MLP	Fusarium head blight	BRR and GP
20	2020	Waldmann et al. [43]	Pig data	CNN	Trait number of live born piglets	GBLUP, BL
21	2020	Pook et al. [82]	Arabidopsis	MLP and CNN	Arabidopsis traits	GBLUP, EGBLUP, BayesA
23	2020	Pérez-Rodríguez et al. [83]	Maize and wheat	MLP	Leaf spot diseases, Gray Leaf Spot	Bayesian ordered probit linear model

RF denotes random forest. Ordinal least square (OLS), Classical Ridge regression (RR), Classical Lasso Regression (LR) and classic elastic net regression (ER). Bayesian Lasso (BL), DBN denotes deep belief networks. GTB denotes Gradient Tree Boosting. GP denotes generalized Poisson regression. EGBLUP denotes extended GBLUP

### A meta-picture of the prediction performance of DL methods in genomic selection

Gianola et al. [61] found that the MLP outperformed a Bayesian linear model in predictive ability in both datasets, but more clearly in wheat. The predictive Pearson’s correlation in wheat ranged from 0.48 ± 0.03 with the BRR, from 0.54 ± 0.03 for MLP with one neuron, from 0.56 ± 0.02 for MLP with two neurons, from 0.57 ± 0.02 for MLP with three neurons and from 0.59 ± 0.02 for MLP with four neurons. Clear and significant differences between BRR and deep learning (MLP) were observed. The improvements of MLP over the BRR were 11.2, 14.3, 15.8 and 18.6% in predictive performance in terms of Pearson’s correlation for 1, 2, 3 and 4 neurons in the hidden layer, respectively. However, for the Jersey data, in terms of Pearson’s correlations Gianola et al. [61] found that the MLP across the six neurons used in the implementation outperformed the BRR by 52% (with pedigree) and 10% (with markers) in fat yield, 33% (with pedigree) and 16% (with markers) in milk yield, and 82% (with pedigree) and 8% (with markers) in protein yield.

Pérez-Rodríguez et al. [62] compared the predictive ability of Radial Basis Function Neural Networks and Bayesian Regularized Neural Networks against several linear models [BL, BayesA, BayesB, BRR and semi-parametric models based on Kernels (Reproducing Kernel Hilbert Spaces)]. The authors fitted the models using several wheat datasets and concluded that, in general, non-linear models (neural networks and kernel models) had better overall prediction accuracy than the linear regression specification. On the other hand, for maize data sets Gonzalez-Camacho et al. [6] performed a comparative study between the MLP, RKHS regression and BL regression for 21 environment-trait combinations measured in 300 tropical inbred lines. Overall, the three methods performed similarly, with only a slight superiority of RKHS (average correlation across trait-environment combination, 0.553) over RBFNN (across trait-environment combination, 0.547) and the linear model (across trait-environment combination, 0.542). These authors concluded that the three models had very similar overall prediction accuracy, with only slight superiority of RKHS and RBFNN over the additive Bayesian LASSO model.

**Fig. 5:**
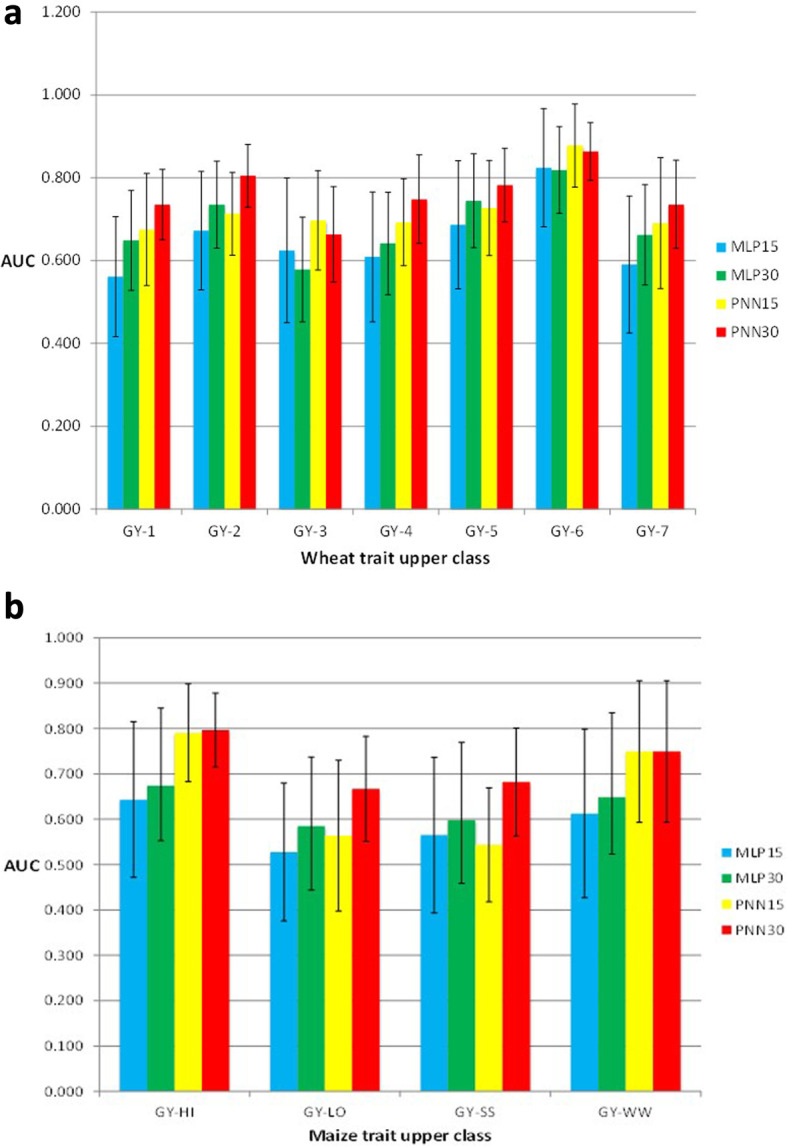
Histograms of the *AUC* criterion and their standard deviation (error bars) for the wheat (**a**) and maize (**b**) datasets. **a**: grain yield (GY) in seven environments (1–7) of classifiers MLP and PNN of the upper 15 and 30% classes; **b**: grain yield (GY) under optimal conditions (HI and WW) and stress conditions (LO and SS) of classifiers MLP and PNN in the upper 15 and 30% classes

Ehret et al. [63], using data of Holstein-Friesian and German Fleckvih cattle, compared the GBLUP model versus the MLP (normal and best) and found non-relevant differences between the two models in terms of prediction performance. In the German Fleckvieh bulls dataset, the average prediction performance across traits in terms of Pearson’s correlation was equal to 0.67 (in GBLUP and MLP best) and equal to 0.54 in MLP normal. In Holstein-Friesian bulls, the Pearson’s correlations across traits were 0.59, 0.51 and 0.57 in the GBLUP, MLP normal and MLP best, respectively, while in the Holstein-Friesian cows, the average Pearson’s correlations across traits were 0.46 (GBLUP), 0.39 (MLP normal) and 0.47 (MLP best). Furthermore, Gonzalez-Camacho et al. [64] studied and compared two classifiers, MLP and probabilistic neural network (PNN). The authors used maize and wheat genomic and phenotypic datasets with different trait-environment combinations. They found that PNN was more accurate than MLP. Results for the wheat dataset with continuous traits split into two and three classes showed that the performance of PNN with three classes was higher than with two classes when classifying individuals into the upper categories (Fig. 5a). Depending on the maize trait-environment combination, the area under the curve (*AUC)* criterion showed that PNN30% or PNN15% upper class (trait grain yield, GY) was usually larger than the *AUC* of MLP; the only exception was PNN15% for GY-SS (Fig. 5b), which was lower than MLP15%.

McDowell [65] compared some conventional genomic prediction models (OLS, RR, LR, ER and BRR) with the MLP in data of Arabidopsis, maize and wheat (Table 2A). He found similar performance between conventional genomic prediction models and the MLP, since in three out of the six traits, the MLP outperformed the conventional genomic prediction models (Table 2A). Based on Pearson’s correlation, Rachmatia et al. [66] found that DL (DBN = deep belief network) outperformed conventional genomic prediction models (RKHS, BL, and GBLUP) in only 1 out of 4 of the traits under study, and across trait-environment combinations, the BL outperformed the other methods by 9.6% (RKHS), 24.28% (GBLUP) and 36.65% (DBN).

**Table 2:** Prediction performance in terms of Pearson’s correlation reported by McDowell (2016); A). Prediction performance in terms of Pearson’s correlation reported by Bellot et al. (2018); B) for traits height and heel bone mineral density. In set “BEST,” the 10 k or 50 k were chosen the top most-associated SNPs, with k = 1000, with the lowest *P*-values in a GWAS on the TRN set for each trait. In set “UNIF,” the genome was split in windows of equal physical length and the most associated SNP within each window was chosen. MLP denotes multilayer perceptron and CNN convolutional neural networks

A	Species	Trait	OLS	RR	LR	ER	BRR	MLP
	Arabidopsis	Dry Matter	0.36	0.4	0.4	0.42	0.39	0.4
		Flowering	0.8	0.82	0.83	0.82	0.82	0.86
	Maize	Flowering	0.22	0.33	0.32	0.33	0.32	0.35
		GY	0.47	0.59	0.49	0.51	0.57	0.55
	Wheat	SGN	0.15	0.27	0.33	0.36	0.28	0.33
		TYM	0.59	0.61	0.74	0.73	0.64	0.76
**B**	**Species**	**Trait**	**Method**	**10kBEST**	**10kUNIF**	**50kBEST**	**50kUNIF**	
	Human	Height	BayesB	0.47	0.38	0.48	0.42	
		Height	BRR	0.47	0.37	0.47	0.39	
		Height	MLP	0.45	0.36	0.45	0.39	
		Height	CNN	0.44	0.34	0.42	0.29	
		HBMD	BayesB	0.28	0.22	0.26	0.24	
		HBMD	BRR	0.28	0.21	0.24	0.22	
		HBMD	MLP	0.15	0.11	0.07	0.09	
		HBMD	CNN	0.27	0.18	0.10	0.11	

*SGN* spike grain number; *TYM* Time young microspore and *HBMD* Heel bone mineral density

Convolutional neural network topology were used by Ma et al. [67] to predict phenotypes from genotypes in wheat and found that the DL method outperformed the GBLUP method. These authors studied eight traits: grain length (GL), grain width (GW), grain hardness (GH), thousand-kernel weight (TKW), test weight (TW), sodium dodecyl sulphate sedimentation (SDS), grain protein (GP), and plant height (PHT). They compared CNN and two popular genomic prediction models (RR-BLUP and GBLUP) and three versions of the MLP [MLP1 with 8–32–1 architecture (i.e., eight nodes in the first hidden layer, 32 nodes in the second hidden layer, and one node in the output layer), MLP2 with 8–1 architecture and MLP3 with 8–32–10–1 architecture]. They found that the best models were CNN, RR-BLUP and GBLUP with Pearson’s correlation coefficient values of 0.742, 0.737 and 0.731, respectively. The other three GS models (MLP1, MLP2, and MLP3) yielded relatively low Pearson’s correlation values, corresponding to 0.409, 0.363, and 0.428, respectively. In general, the DL models with CNN topology were the best of all models in terms of prediction performance.

**Fig. 6:**
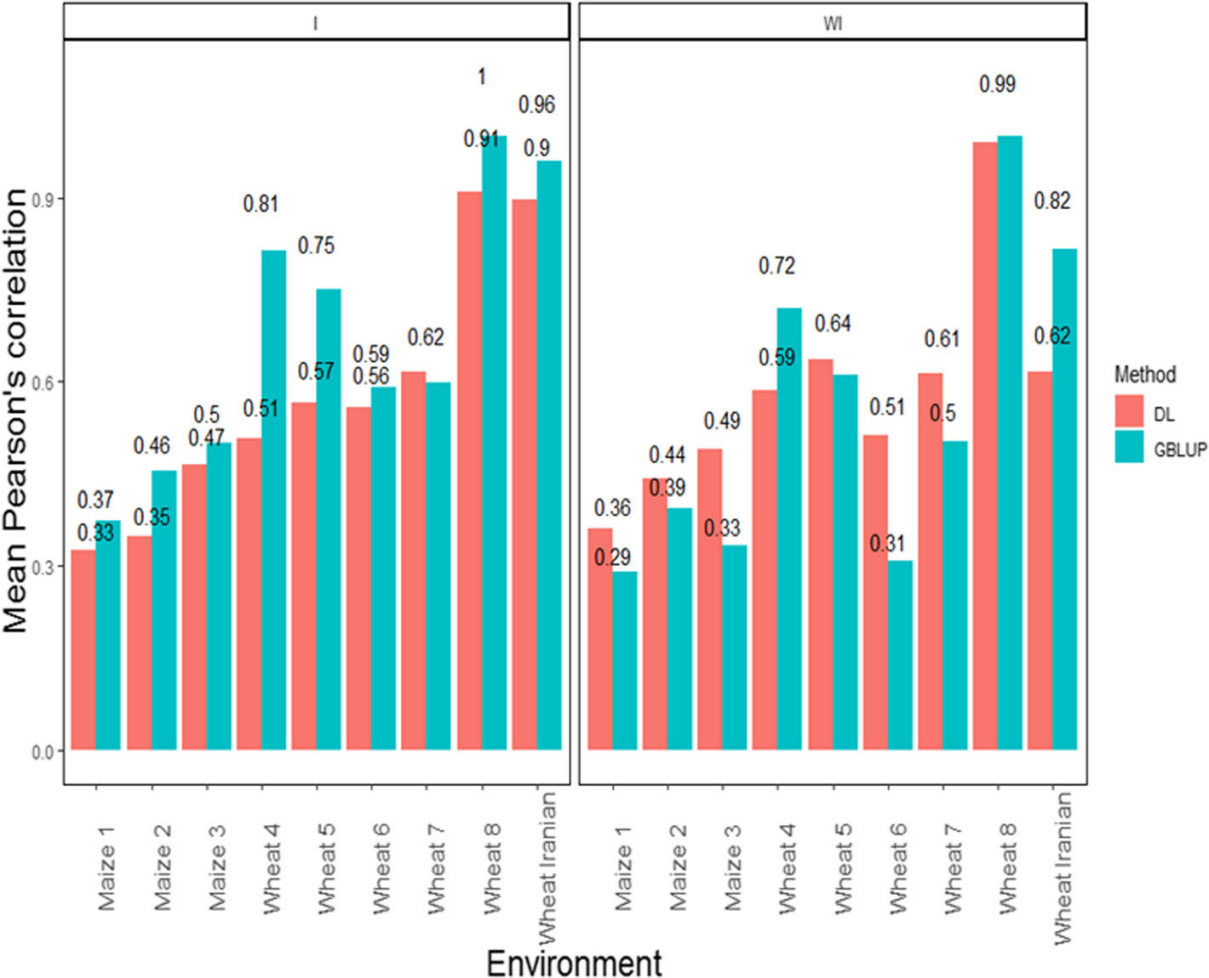
Pearson’s correlation across environments for the GBLUP and the DL model. The first vertical sub-panel corresponds to the model with genotype × environment interaction (I), and the second vertical sub-panel corresponds to the same model but without genotype × environment interaction (WI) (Montesinos-López et al., 2018a)

Waldmann [68] found that the resulting testing set MSE on the simulated TLMAS2010 data were 82.69, 88.42, and 89.22 for MLP, GBLUP, and BL, respectively. Waldmann [68] used Cleveland pig data [69] as an example of real data and found that the test MSE estimates were equal to 0.865, 0.876, and 0.874 for MLP, GBLUP, and BL, respectively. The mean squared error was reduced by at least 6.5% in the simulated data and by at least 1% in the real data. Using nine datasets of maize and wheat, Montesinos-López et al. [70] found that when the G ×*E* interaction term was not taken into account, the DL method was better than the GBLUP model in six out of the nine datasets (see Fig. 6). However, when the G ×*E* interaction term was taken into account, the GBLUP model was the best in eight out of nine datasets (Fig. 6).

Next we compared the prediction performance in terms of Pearson’s correlation of the multi-trait deep learning (MTDL) model versus the Bayesian multi-trait and multi-environment (BMTME) model proposed by Montesinos-López et al. [71] in three datasets (one of maize and two of wheat). These authors found that when the genotype × environment interaction term was not taken into account in the three datasets under study, the best predictions were observed under the MTDL model (in maize BMTME = 0.317 and MTDL = 0.435; in wheat BMTME = 0.765, MTDL = 0.876; in Iranian wheat BMTME = 0.54 and MTDL = 0.669) but when the genotype × environment interaction term was taken into account, the BMTME outperformed the MTDL model (in maize BMTME = 0.456 and MTDL = 0.407; in wheat BMTME = 0.812, MTDL = 0.759; in Iranian wheat BMTME = 0.999 and MTDL = 0.836).

Bellot et al. [72], in a study conducted on complex human traits (height and heel bone mineral density), compared the predictive performance of MLP and CNN with that of Bayesian linear regressions across sets of SNPs (from 10 k to 50 k, with k = 1000) that were preselected using single-marker regression analyses. For height and heel bone mineral density, all methods performed similarly, but in general CNN was the worst. The performance of MLP was highly dependent on SNP set and phenotype. The authors found in general terms that CNN performance was competitive with that of linear models, but they did not find any case where DL outperformed the linear model by a sizable margin (Table 2B).

In another study, Montesinos-López et al. [73] performed a benchmark study to compare univariate deep learning (DL), the support vector machine and the conventional Bayesian threshold best linear unbiased prediction (TGBLUP). They did not find large differences between the three methods. However, in many cases the TGBLUP outperformed the other two methods. The best prediction performance with the interaction term (I) was with the TGBLUP model, with gains of 17.15% (DTHD), 16.11% (DTMT) and 4.64% (Height) compared to the SVM method, and gains of 10.70% (DTHD), 8.20% (DTMT) and 3.11% (Height) compared to the DL model. Without the interaction term (WI), no statistical differences were found between the three methods (TGBLUP, SVM and DL) for the three traits under study. Finally, when comparing the best predictions of the TGBLUP model that were obtained with the genotype × environment interaction (I) term and the best predictions of the SVM and DL models that were obtained without (WI) the interaction term, we found that the TGBLUP model outperformed the SVM method by 1.90% (DTHD), 2.53% (DTMT) and 1.47% (Height), and the DL method by 2.12% (DTHD), 0.35% (DTMT) and 1.07% (Height).

Montesinos-López et al. [74], in a study of durum wheat where they compared GBLUP, univariate deep learning (UDL) and multi-trait deep learning (MTDL), found that when the interaction term (I) was taken into account, the best predictions in terms of mean arctangent absolute percentage error (MAAPE) across trait-environment combinations were observed under the GBLUP (MAAPE = 0.0714) model and the worst under the UDL (MAAPE = 0.1303) model, and the second best under the MTDL (MAAPE = 0.094) method. However, when the interaction term was ignored, the best predictions were observed under the GBLUP (MAAPE = 0.0745) method and the MTDL (MAAPE = 0.0726) model, and the worst under the UDL (MAAPE = 0.1156) model; non-relevant differences were observed in the predictions between the GBLUP and MTDL.

Khaki and Wang [75], in a maize dataset of the 2018 Syngenta Crop Challenge, evaluated the prediction performance of the MLP (deep learning) method against the performance of Lasso regression and regression tree. The training set consisted of 2267 maize hybrids planted in 2247 locations between 2008 and 2016, and the participants were asked to predict (testing set) the yield performance in 2017. They predicted grain yield, check yield (average yield across all hybrids of the same location) and the yield difference. The yield difference was the difference between the grain yield and the check yield, and indicated the relative performance of a hybrid against other hybrids at the same location [76]. They found that in general the MLP model with 20 hidden layers outperformed conventional genomic prediction models (LR and RT) and also the MLP model with only one hidden layer (SNN) (Table 3A), but the best performance was observed in the GY trait.

**Table 3:** Prediction performance in terms of root mean square error of prediction (RMSE) of the four models (MLP_20, LR, MLP_1, RT) reported by Khaki and Wang (2019); A) in a maize dataset. MPL_20 denotes the MLP model with 20 hidden layers, and MPL_1 denotes the MLP model with 1 hidden layer. Prediction performance in terms of Pearson’s correlation of 6 species across traits evaluated with 11 methods (Azodi et al., 2019); B). SVR denotes support vector regression. SVR_lin denotes SVR with linear kernel, SVR_poly denotes SVR with polynomial kernel, SVR_rbf denotes SVR with kernel Radial Basis Function

A		Model	Trait		RMSE		
		MLP_20	Yield		12.79		
			Check yield		11.38		
			Yield difference		12.4		
		LR	Yield		21.4		
			Check yield		19.87		
			Yield difference		13.11		
		MLP_1	Yield		18.04		
			Check yield		15.18		
			Yield difference		15.19		
		RT	Yield		15.03		
			Check yield		14.87		
			Yield difference		15.92		
**B**	**Method**	**Maize**	**Rice**	**Sorghum**	**Soy**	**Spruce**	**Switch-grass**
	rrBLUP	0.44	0.34	0.63	0.46	0.32	0.61
	BRR	0.44	0.39	0.63	0.46	0.32	0.61
	BayesA	0.42	0.38	0.63	0.47	0.32	0.61
	BayesB	0.43	0.38	0.63	0.46	0.32	0.61
	BL	0.44	0.39	0.62	0.46	0.32	0.61
	SVR_lin	0.41	0.38	0.62	0.43	0.19	0.6
	SVR_poly	0.43	0.38	0.63	0.41	0.33	0.61
	SVR_rbf	0.39	0.38	0.63	0.04	0.34	0.6
	RF	0.43	0.4	0.58	0.36	0.35	0.57
	GTB	0.37	0.38	0.58	0.4	0.33	0.56
	MLP	0.17	0.08	0.45	0.44	0.28	0.45

Azodi et al. [77], using data of six plant species and 18 traits across different training population sizes and marker densities, compared the performance of six linear and five non-linear machine learning models, including DL models. They concluded that across all traits and species, no one algorithm performed best; however, predictions based on a combination of results from multiple algorithms (i.e., ensemble predictions) performed consistently well. While linear and non-linear algorithms performed best for a similar number of traits, the performance of non-linear algorithms varied more between traits than that of linear algorithms (Table 3B). On the other hand, the results of Liu et al. [78] in soybean show that DL models outperformed conventional genomic prediction models (rrBLUP, BRR, BayesA, BL) using Pearson’s correlation as a metric. Among the deep learning models in three of the five traits, the MLP model outperformed the other DL methods (dualCNN, deepGS and singleCNN) (Table 4A).

**Table 4:** Prediction performance in soybean for five traits of eight methods in terms of Pearson’s correlation (taken from Liu et al., 2019); A). Methods dualCNN, deepGS and singleCNN are different versions of CNN. Prediction performance in terms of Average Spearman Correlation (ASC) and mean square error (MSE) with genotype × environment interaction (I) and without genotype × environment interaction (WI) in a wheat dataset for trait Fusarium head blight (FHB) severity data (Montesinos-López et al., 2020; B)

A	Method	Yield	Protein	Oil	Moisture	Height
	dualCNN	0.452	0.619	0.668	0.463	0.615
	DeepGS	0.391	0.506	0.531	0.31	0.452
	Dense	0.449	0.603	0.657	0.427	0.612
	singleCNN	0.463	0.573	0.627	0.449	0.565
	rrBLUP	0.412	0.392	0.39	0.413	0.458
	BRR	0.422	0.392	0.39	0.413	0.458
	Bayes A	0.419	0.393	0.388	0.415	0.458
	BL	0.419	0.394	0.388	0.416	0.458
**B**	**Interaction**	**Type**	**ASC**	**SE**	**MSE**	**SE**
	I	BRR	0.584	0.012	3.015	0.169
	I	NDNN	0.626	0.013	1.891	0.088
	I	GP	0.596	0.01	2.457	0.121
	I	PDNN	0.627	0.012	1.912	0.073
	WI	BRR	0.436	0.018	4.481	0.25
	WI	NDNN	0.635	0.013	1.872	0.084
	WI	GP	0.431	0.018	3.418	0.186
	WI	PDNN	0.584	0.014	2.853	0.412

Abdollahi-Arpanahi et al. [79] conducted a study related to the sire conception rate (SCR) of 11,790 Holstein bulls genotyped with 58 k single nucleotide polymorphisms (SNPs). In terms of mean square error of prediction, they reported that the best prediction performance was observed in the gradient boosting method (3.976), followed by Bayes B (4.036), GBLUP (4.049), RF (4.186), CNN (4.269) and MLP (4.428). A similar pattern was observed in terms of average Pearson’s correlation where the boosting method was the best (0.358), followed by Bayes B (0.338), GBLUP (0.333), RF (0.316), CNN (0.291) and MLP (0.264).

In strawberry and blueberry, Zingaretti et al. [80] also compared conventional genomic prediction models (BL, BRR, BRR-GM and RKHS) with CNNs (a type of DL model). They found in real datasets that when averaged across traits in the strawberry species, prediction accuracies in terms of average Pearson’s correlation were 0.43 (BL), 0.43 (BRR), 0.44 (BRR-GM), 0.44 (RKHS), and 0.44 (CNN). By trait, the BRR-GM was best in average fruit weight prediction, BL, BRR, and RKHS were best for early marketable yield, and RKHS and BRR-GM for total marketable weight, whereas CNN performed the best in soluble solid content and percentage of culled fruit [80]. In general, these authors found that linear Bayesian models were better than convolutional neural networks for the full additive architecture, whereas the opposite was observed under strong epistasis. For blueberry, these authors [80] did not find statistical differences between BL and BRR (average Pearson’s correlation: 0.42), but these two Bayesian methods outperformed CNNs (average Pearson’s correlation: 0.40).

Montesinos-López et al. [81] report that the best performance in terms of Average Spearman Correlation (ASC) occurred under the deep learning models [normal deep neural network (NDNN) and Poisson deep neural network (PDNN)], while the worst was under the Bayesian (BRR) and classic generalized Poisson model (GP) (Table 4B). However, Table 4B also shows that without genotype × environment interaction (WI), the NDNN models were better than the PDNN models, but when taking WI into account, no differences were observed between these deep learning models. They also found that the PDNN model outperformed the GP model by 5.20% (in terms of ASC) under I, and 35.498% (in terms of ASC) under WI. With regard to the BRR model, the PDNN model was superior by 7.363% (in terms of ASC) under I, and by 33.944% (in terms of ASC) under WI. The same behavior is observed in Table 4B under the MSE metrics, where we can see that the deep learning models were the best, but without the genotype × environment interaction, the NDNN models were slightly better than the PDNN models.

Waldmann et al. [43] also used the TLMAS2010 data from the Waldmann et al. [68] article and found that under the CNN, the MSE was equal to 62.34 while the GBLUP and BL produced mean MSE over folds of 88.42 and 89.22, respectively. This implies that the improvement for the simulated data was 29.5 and 30.1%, respectively. Under the real pig dataset [69], the observed MSE were 3.51, 3.64 and 3.61 for the CNN, GBLUP and BL models, respectively; this means that CNN gained only 3.57% over the GBLUP and only 2.78% over the BL model [43]. Pook et al. [82] found that in the simulated dataset, local CNN (LCNN) outperformed conventional CNN, MLP, GBLUP, BNN, BayesA, and EGLUP (Table 5A). However, with the real Arabidopsis dataset, the prediction performance of the DL models (MLP, CNN and LCNN) was slightly worse than that of conventional genomic prediction models (GBLUP, BayesA and EGBLUP) (Table 5B).

**Table 5:** Prediction performance in terms of Pearson’s correlation for the simulated and real Arabidopsis datasets (Pook et al., 2020)

A). Predictive ability on different traits with						
Trait architecture	GBLUP	BayesA	EGBLUP	MPL	CNN	LCNN
10 additive QTL	0.639	0.66	0.635	0.637	0.627	0.666
1000 additive QTL	0.516	0.538	0.543	0.524	0.538	0.606
10 epistatic QTL	0.511	0.527	0.519	0.503	0.491	0.572
1000 epistatic QTL	0.416	0.414	0.448	0.395	0.403	0.401
10 locally linked epistatic QTL	0.488	0.501	0.529	0.504	0.544	0.625
1000 locally linked epistatic QTL	0.524	0.523	0.541	0.519	0.517	0.51
**B). Predictive ability for the Arabidopsis traits**						
Trait architecture	GBLUP	BayesA	EGBLUP	MLP	CNN	LCNN
Average predictive ability (all)	0.39	0.382	0.382	0.316	0.312	0.34
Average predictive ability (training set < 100)	0.404	0.39	0.399	0.3	0.299	0.326
Average predictive ability (100 < training set < 250)	0.364	0.358	0.354	0.318	0.311	0.327
Average predictive ability (training set > 250)	0.477	0.477	0.472	0.358	0.37	0.456

A neural network for modeling ordinal data using a data augmentation approach was proposed by Pérez-Rodríguez et al. [83]. The authors proposed using the Generalized EM algorithm. The predictive ability of the proposed model was tested using two datasets: 1) Septoria, a fungus that causes leaf spot diseases in field crops, forage crops and vegetables which was evaluated on CIMMYT wheat lines; and 2) Gray Leaf Spot, a disease caused by the fungus *Cercospora zeae-maydis* for maize lines from the Drought Tolerance maize program at CIMMYT. The authors evaluated several performance measures (Brier Score, Missclassification Error Rate, Mean Absolute Error, Spearman correlation coefficient) and concluded that in general the proposed neural network had better performance than the Bayesian ordered probit linear model that is widely used in ordinal data analysis.

### Pros and cons of DL methods

Even with few applications in GS, DL models are attractive and promising tools for the following reasons: (a) DL models naturally capture, without the need to specify additional terms in the predictor (like interactions), non-additive effects and complex relationships and interactions in large datasets, which is key for capturing the whole genetic merit; (b) they efficiently handle not only large data, but also raw data like images without any preprocessing (feature engineering not required); for this reason, DL models more efficiently incorporate large numbers of omics data (Metabolomics, microbiomics, phenomics, Proteomics, Transcriptomics, etc.) in the same model, which is not possible with most machine learning and statistical learning methods; (c) frameworks for DL are very flexible because their implementation allows training models with continuous, binary, categorical and count outcomes, with many hidden layers (1,2, …), many types of activation functions (RELU, leakyRELU, sigmoid, etc.), many optimizers (Adam, sgd, rmsprop, adagrad, adadelta, adamax, nadam), and many latent variables by using autoencoder or embedding as a generative latent variable model, many topologies that can capture very complex linear and nonlinear patterns in the data, and allows many types of inputs (images, numbers, etc.); (d) there is much empirical evidence that the larger the dataset, the better the performance of DL models, which offers many opportunities to design specific topologies (deep neural networks) to deal with any type of data in a better way than current models used in GS, because DL models with topologies like CNN can very efficiently capture the correlation (special structure) between adjacent input variables, that is, linkage disequilibrium between nearby SNPs; (f) some DL topologies like CNN have the capability to significantly reduce the number of parameters (number of operations) that need to be estimated because CNN allows sharing parameters and performing data compression (using the pooling operation) without the need to estimate more parameters; and (g) the modeling paradigm of DL is closer to the complex systems that give rise to the observed phenotypic values of some traits. For these reasons, the incorporation of DL for classical breeding pipelines is in progress and some uses of DL are given next: 1) for the prediction of parental combinations, which is critical for choosing superior combinational homozygous parental lines in F1-hybrid breeding programs [84], 2) for modelling and predicting quantitative characteristics, for example, to perform image-based ear counting of wheat with high level of robustness, without considering variables, such as growth stage and weather conditions [85], 3) for genetic diversity and genotype classification, for example, in *Cinnamomum osmophloeum* Kanehira (Lauraceae), DL was applied to differentiate between morphologically similar species [86], and 4) for genomic selection (see Table 1).

Because DL has many advantages, it is extremely popular and its applications are everywhere. Nevertheless, DL is not a panacea since it is not the best option in all types of problems; some of the caveats of this DL methodology for GS are: (a) it is not really useful for inference and association studies, since its parameters (weights) many times cannot be interpreted as in many statistical models; also, since neither feature selection nor feature importance is obvious, for this reason, the DL methodology inhibits testing hypotheses about the biological meaning with the parameter estimates; (b) when studying the association of phenotypes with genotypes, it is more difficult to find a global optimum, since the loss function may present local minima and maxima; (c) these models are more prone to overfitting than conventional statistical models mostly in the presence of inputs of large dimensions, since to efficiently learn the pattern of the data, more hidden layers and neurons need to be taken into account in the DL models; however, there is evidence that these problems can be solved under a Bayesian approach and some research is going in this direction to implement DL models under a Bayesian paradigm [87]; but two of the problems under the Bayesian framework are how to elicit priors and the fact that considerably more computational resources are required; (d) considerable knowledge is required for implementing appropriate DL models and understanding the biological significance of the outputs, since this requires a very complex tuning process that depends on many hyper-parameters; (e) although there is very user-friendly software (Keras, etc.) for DL, its implementation is very challenging since it depends strongly on the choice of hyper-parameters, which requires a considerable amount of time and experience and, of course, considerable computational resources [88, 89]; (f) DL models are difficult to implement in GS because genomic data most of the time contain more independent variables than samples (observations); and (g) another disadvantage of DL is the generally longer training time required [90].

### Trends of DL applications

In the coming 10 years, DL will be democratized via every software-development platform, since DL tools will incorporate simplified programming frameworks for easy and fast coding. However, as automation of DL tools continues, there’s an inherent risk that the technology will develop into something so complex that the average users will find themselves uninformed about what is behind the software.

Nowadays, unsupervised methods (where you only have independent variables [input] but not dependent variables [outcomes]) are quite inefficient, but it is expected that in the coming years, unsupervised learning methods will be able to match the “accuracy and effectiveness” of supervised learning. This jump will dramatically reduce the cost of implementing DL methods, which now need large volumes of labeled data with inputs and outputs. In the same direction, we expect the introduction of new DL algorithms that will allow testing hypotheses about the biological meaning with parameter estimates (good for inference and explainability), that is, algorithms that are not only good for making predictions, but also useful for explaining the phenomenon (*actual functional biology of the phenotype***)** to increase human understanding (or knowledge) of complex biological systems.

In the coming years, we expect a more fully automated process for learning and explaining the outputs of implemented DL and machine learning models. This means that it is feasible to develop systems that can automatically discover plausible models from data, and explain what they discovered; these models should be able, not only to make good predictions, but also to test hypotheses and in this way unravel the complex biological systems that give rise to the phenomenon under study.

### General considerations

GS as a predictive tool is receiving a lot of attention in plant breeding since it is powerful for selecting candidate individuals early in time by measuring only genotypic information in the testing set and both phenotypic and genotypic information in the training set. For this reason, this predictive methodology has been adopted for crop improvement in many crops and countries. GS can perform the selection process more cheaply and in considerably less time than conventional breeding programs. This will be key for significantly increasing the genetic gain and reducing the food security pressure since we will need to produce 70% more food to meet the demands of 9.5 billion people by 2050 [1]. Thanks to the ever-increasing data generated by industry, farmers, and scholars, GS is expected to improve efficiency and help make specific breeding decisions. For this reason, a wide range of analytical methods, such as machine learning, deep learning, and artificial intelligence, are now being adapted for application in plant breeding to support analytics and decision-making processes [91].

The prediction performance in GS is affected by the size of the training dataset, the number of markers, the heritability, the genetic architecture of the target trait, the degree of correlation between the training and testing set, etc. Deep learning can be really powerful for prediction if used appropriately, and can help to more efficiently map the relationship between the phenotype and all inputs (markers, all remaining omics data, imaginary data, geospatial and environmental variables, etc.) to be able to address long-standing problems in GS in terms of prediction efficiency.

We found that DL has impressive potential to provide good prediction performance in genomic selection. However, there is not much evidence of its utility for extracting biological insights from data and for making robust assessments in diverse settings that might be different from the training data. Beyond making predictions, deep learning could become a powerful tool for synthetic biology by learning to automatically generate new DNA sequences and new proteins with desirable properties.

However, more iterative and collaborative experimentation needs to be done to be able to take advantage of DL in genomic selection. In terms of experimentation, we need to design better strategies to better evaluate the prediction performance of genomic selection in field experiments that are as close as possible to real breeding programs. In terms of collaborative work, we need to strengthen interdisciplinary work between breeders, biometricians, computer scientists, etc., to be able to automatically collect (record) more data, the costs of which continue to decrease. The data should include not only phenotypic data, but also many types of omics data (metabolomics, microbiomics, phenomics using sensors and high resolution imagery, proteomics, transcriptomics, etc.), geoclimatic data, image data from plants, data from breeders’ experience, etc., that are high quality and representative of real breeding programs. Then, with all collected data, we need to design efficient topologies of DL models to improve the selection process of candidate individuals. This is feasible because DL models are really powerful for efficiently combining different kinds of inputs and reduce the need for feature engineering (FE) the input. FE is a complex, time-consuming process which needs to be altered whatever the problem. Thus, FE constitutes an expensive effort that is data dependent and requires experts’ knowledge and does not generalize well [92]. However, this is an iterative process (with trial and error) where all the members of this network (breeders, biometricians, computer scientists, molecular biologists, etc.) need to contribute their knowledge and experience to reach the main goal. In this way, it is very likely that the process of selecting candidate individuals with GS will be better than the conventional selection process. For example, before 2015, humans were better than artificial machines at classifying images and solving many problems of computer vision, but now machines have surpassed the classification ability of humans, which was considered impossible only some years ago. In 2016, a robot player beat a human player in the famed game AlphaGo, which was considered an almost impossible task. DL also outperformed 136 of 157 dermatologists in a head-to-head dermoscopic melanoma image classification task [93].

However, this task of DL (i.e., selecting the best candidate individuals in breeding programs) requires not only larger datasets with higher data quality, but also the ability to design appropriate DL topologies that can combine and exploit all the available collected data. This is important since the topologies designed for computer vision problems are domain specific and cannot be extrapolated straightforwardly to GS. For example, in GS most of the time the number of inputs is considerably larger than the number of observations, and the data are extremely noisy, redundant and with inputs of different origins. However, since intelligence relies on understanding and acting in an imperfectly sensed and uncertain world, there is still a lot of room for more intelligent systems that can help take advantage of all the data that are now being collected and make the selection process of candidate individuals in GS extremely more efficient.

We found no relevant differences in terms of prediction performance between conventional genome-based prediction models and DL models, since in 11 out of 23 studied papers (see Table 1), DL was best in terms of prediction performance taking into account the genotype by interaction term; however, when ignoring the genotype by environment interaction, DL was better in 13 out of 21 papers. This in part is explained by the fact that not all data contain nonlinear patterns, not all are large enough to guarantee a good learning process, were tuned efficiently, or used the most appropriate architecture (examples: shallow layers, few neurons, etc.); in addition, the design of the training-tuning-testing sets may not have been optimal, etc. However, we observed that most of the papers in which the DL models outperformed conventional GS models were those in which different versions of CNN were used. There is also a lot of empirical evidence that CNN are some of the best tools for prediction machines when the inputs are raw images. Some experts attribute the many successful commercial applications of DL (which most of the time reach or exceed human performance level) to the building and improvement of this type of topologies that in part are also responsible for the term deep learning coined to denote artificial neural networks with more than one hidden layer. CNNs are different than MLP because they are able to more efficiently capture spatial structure patterns that are common in image inputs. For this reason, CNNs are being very successfully applied to complex tasks in plant science for: (a) root and shoot feature identification [94], (b) leaf counting [95, 96], (c) classification of biotic and abiotic stress [97], (d) counting seeds per pot [98], (e) detecting wheat spikes [99], and (f) estimating plant morphology and developmental stages [100], etc. These examples show that DL is playing an important role in obtaining better phenotypes in the field and indirectly affects genomic prediction performance. Although DL does not always outperform conventional regression methods, these examples show that DL is accelerating the progress in prediction performance, and we are entering a new era where we will be able to predict almost anything given good inputs.

In this contribution, we attempt to clarify issues that have being preventing the use of DL methods at the breeding level, for instance, that DL is a complete “black box” methodology, without much statistical fundamentals. There is a widespread sense that implementing DL into a breeding pipeline is not straightforward without a strong statistical/computing background associated to the use of super computers - both limiting factors for some modest breeding programs. Although the learning curve for DL can be slow, in the **Appendix** we show a maize toy example with 5 folds cross validation.

Finally, since nowadays GS requires high-throughput genotyping systems and biometrics expertise that might not easily accessible to breeding programs in developing countries, increased sharing of genomic resources, genomic data, quantitative genetics and biometrics expertise between developed countries, developing regions and emerging economies will be the key to global food security in an era of rapid climate and environmental change.

## Conclusions

Deep learning is a disruptive technology that has immense potential for applications in any area of predictive data science. An essential requirement is the availability of high quality and sufficiently large training data. However, based on the considered publications on the use of DL for genomic selection, we did not find strong evidence for its clear superiority in terms of prediction power compared to conventional genomic prediction models. We obtained evidence that DL algorithms are powerful for capturing nonlinear patterns more efficiently than conventional genomic prediction models and for integrating data from different sources without the need for feature engineering. DL algorithms also have a lot of potential to improve prediction accuracy by developing specific topologies for the type of data at hand in plant breeding programs. For these reasons, it is of paramount importance to adopt this disruptive technology in GS and be able to increase its efficiency and accuracy. However, DL models cannot be adopted for GS blindly in small training-testing data sets.

## Data Availability

Not applicable.

## Appendix

### Maize toy example with 5 folds CV with only one inner CV

rm. (list = ls()).

library (lsa).

library (keras).

library (BMTME).

library (plyr).

library (tidyr).

library (dplyr).

##########Set seed for reproducible results###################.

use_session_with_seed(45).

#################Loading the MaizeToy Datasets###############.

data(“MaizeToy”).

head (phenoMaizeToy).

###########Ordering the data ################################.

phenoMaizeToy<−(phenoMaizeToy [order (phenoMaizeToy$Env,phenoMaizeToy$Line),])

rownames (phenoMaizeToy)=1:nrow (phenoMaizeToy).

head (phenoMaizeToy,8).

#################Design matrices############### #####################.

LG- cholesky (genoMaizeToy).

ZG- model.matrix(~0+as.factor (phenoMaizeToy$Line)).

Z.G- ZG %*%LG.

Z.E- model.matrix(~0+as.factor (phenoMaizeToy$Env)).

ZEG < - model.matrix(~ 0 + as.factor (phenoMaizeToy$Line):as.factor (phenoMaizeToy$Env)).

G2 < - kronecker (diag (length (unique (phenoMaizeToy$Env))), data.matrix (genoMaizeToy)).

LG2 < - cholesky(G2).

Z.EG < - ZEG %*% LG2.

############Selecting the response variable#######################.

Y < - as.matrix (phenoMaizeToy[, −c(1, 2)]).

####Training testing sets using the BMTME package###############.

pheno <− data.frame (GID = phenoMaizeToy[, 1], Env= phenoMaizeToy[, 2],

Response=phenoMaizeToy[, 3]).

CrossV <− CV.KFold (pheno, DataSetID = ‘GID’, K=5, set_seed=123).

#########Grid of hyperparameters######## ############# #####################.

Stage <− expand.grid (units_M=seq(33,67,33),epochs_M = 1000, Dropout = c(0.0,0.05,0.15, 0.25, 0.35)).

################Function for averaging the predictions############.

summary. BMTMECV <- function (results, information = ‘compact’, digits = 4, ...) {.

results % > %.

group_by(Environment, Trait, Partition) % > %.

summarise (MSE = mean((Predicted-Observed)^2),

MAAPE = mean (atan (abs (Observed-Predicted)/abs (Observed)))) % > %.

select (Environment, Trait, Partition, MSE, MAAPE) % > %.

mutate_if(is.numeric, funs (round(., digits))) % > %.

as.data.frame() - > presum.

presum % > % group_by(Environment, Trait) % > %.

summarise (SE_MAAPE = sd (MAAPE, na.rm. = T)/sqrt(n()), MAAPE = mean (MAAPE, na.rm. = T),

SE_MSE = sd (MSE, na.rm. = T)/sqrt(n()), MSE = mean (MSE, na.rm. = T)) % > %.

select (Environment, Trait, MSE, SE_MSE, MAAPE, SE_MAAPE) % > %.

mutate_if(is.numeric, funs (round(., digits))) % > %.

as.data.frame() - > finalSum.

out <− switch (information,

compact = finalSum,

complete = presum,

extended = {.

finalSum$Partition <− ‘All’.

presum$Partition <− as.character (presum$Partition).

presum$SE_MSE < - NA.

presum$SE_MAAPE <- NA.

rbind (presum, finalSum).

}

)

return (out).

}

#######Final X and y for fitting the model###################.

y = (phenoMaizeToy[, 3]).

X = cbind(Z.E,Z.G).

############Outer Cross-validation#######################.

digits = 4.

Names_Traits = colnames(Y).

results = data.frame().

t = 1.

for (o in 1:5){.

tst_set = CrossV$CrossValidation_list[[o]].

X_trn = (X[−tst_set,])

X_tst = (X [tst_set,])

y_trn = (y[−tst_set]).

y_tst = (y [tst_set]).

nCVI = 1 ####Number of folds for inner CV.

i = 1.

#########Matrices for saving the output of inner CV#######################.

Tab_pred_MSE = matrix (NA,ncol = length (Stage[,1]), nrow = nCVI).

Tab_pred_Epoch = matrix (NA,ncol = length (Stage[,1]), nrow = nCVI).

Tab_pred_Units = matrix (NA,ncol = length (Stage[,1]), nrow = nCVI).

Tab_pred_Drop = matrix (NA,ncol = length (Stage[,1]), nrow = nCVI).

X_trI = X_trn.

y_trI = y_trn.

for (stage in seq_len(dim (Stage) [1])) {.

X_trII = X_trI.

y_trII = y_trI.

units_M < - Stage [stage, 1].

epochs_M < - Stage [stage, 2].

Drop_per = Stage [stage, 3].

build_model<−function() {.

model <− keras_model_sequential().

model % > %.

layer_dense(units = units_M, activation = “relu”, input_shape = c (dim(X_trII) [2])) % > %.

layer_dropout(rate = Drop_per) % > %.

layer_dense(units = 1, activation = “relu”).

model % > % compile(.

loss = “mse”,

optimizer = optimizer_adam(),

metrics = c(“mse”)).

model}.

model<−build_model().

model % > % summary().

print_dot_callback <− callback_lambda(.

on_epoch_end = function (epoch, logs) {.

if (epoch %% 20 == 0) cat(“\n”).

cat(“.”)

})

########Fitting the model with Early stopping#######.

early_stop <− callback_early_stopping(monitor = “val_loss”,mode = ‘min’,patience =50).

###########Fit of the model for each values of the grid#################.

model_Final<−build_model().

model_fit_Final<−model_Final % > % fit(.

X_trII, y_trII,

epochs = epochs_M, batch_size =72,

###shuffled = F,

validation_split = 0.2,

verbose = 0,callbacks = list (early_stop, print_dot_callback)).

###########Saving the output of each hyperparameter###################.

No.Epoch_Min = length (model_fit_Final$metrics$val_mean_squared_error).

Min_MSE = model_fit_Final$metrics$val_mean_squared_error[No.Epoch_Min].

Tab_pred_MSE[i,stage] = Min_MSE.

Tab_pred_Units[i,stage] = units_M.

Tab_pred_Epoch[i,stage] = No.Epoch_Min [1].

Tab_pred_Drop[i,stage] = Drop_per.

}

##############Selecting the optimal hyperparameters##############.

Median_MSE_Inner = apply (Tab_pred_MSE,2,median).

Units_Inner = apply (Tab_pred_Units,2,max).

Drop_Inner = apply (Tab_pred_Drop,2,max).

Epoch_Inner = apply (Tab_pred_Epoch,2,median).

Pos_Min_MSE = which (Median_MSE_Inner==min (Median_MSE_Inner)).

Units_O=Units_Inner[Pos_Min_MSE].

Epoch_O = Epoch_Inner[Pos_Min_MSE].

Drop_O = Drop_Inner[Pos_Min_MSE].

###########Refitting the model with the optimal values#################.

model_Sec < −keras_model_sequential().

model_Sec % > %.

layer_dense(units = Units_O, activation = “relu”, input_shape = c (dim(X_trn) [2])) % > %.

layer_dropout(rate = Drop_O) % > %.

layer_dense(units =1).

model_Sec % > % compile(.

loss = “mean_squared_error”,

optimizer = optimizer_adam(),

metrics = c(“mean_squared_error”)).

ModelFited <−model_Sec % > % fit(.

X_trn, y_trn,

epochs = Epoch_O, batch_size =30,

####### validation_split = 0.2,early_stop,

verbose = 0, callbacks = list (print_dot_callback)).

####Prediction of testing set ##########################.

Yhat = model_Sec % > % predict(X_tst).

y_p = Yhat.

y_p_tst = as.numeric(y_p).

###########Saving the predicctions of each outer-testing set#################.

results<−rbind (results, data.frame (Position = tst_set,

Environment = CrossV$Environments [tst_set],

Partition = o,

Units = Units_O,

Epochs = Epoch_O,

Drop_Out = Drop_O,

Observed = round(y [tst_set], digits), #$response, digits),

Predicted = round(y_p_tst, digits),

Trait = Names_Traits[t])).

cat(“CV = “,o,”\n”).

}

### Results

#############Average of prediction performance##################################.

Pred_Summary = summary. BMTMECV (results = results, information = ‘compact’, digits = 4).

Pred_Summary.

## Abbreviations

PS: Phenotypic Selection
GS: Genomic Selection
DL: Deep Learning
ML: Machine Learning
GBLUP: Genomic Best Linear Unbiased Predictor
TGBLUP: Threshold Genomic Best Linear Unbiased Predictor
RR-BLUP or rrBLUP: Ridge Regression Best Linear Unbiased Predictor
BMTME: Bayesian multi-trait and multi-environment
DNN: Deep Neural Network
MLP: Multilayer Perceptron
CNN: Convolutional Neural Networks
ReLu: Rectified linear activation unit
BRR: Bayesian Ridge regression
OLS: Ordinary Least Squares
LR: Linear regression
RKHS: Reproducing Kernel Hilbert Spaces
RNN: Recurrent Neural Network
PNN: Probabilistic Neural Network
*AUC*: Area Under the Curve
RBFNN: Kernel Radial Basis Function Neural Network
RF: Random Forest
RR: Classical Ridge Regression
ER: Classical Elastic Net
DBN: Deep Belief Networks
SVR: Support Vector Regression
UDL: Univariate Deep Learning
MTDL: Multivariate Deep Learning
MAAPE: Mean Arctangent Absolute Percentage Error
ASC: Average Spearman Correlation
NDNN: Normal Deep Neural Network
PDNN: Poisson Deep Neural Network,
GP: Classic Generalized Poisson model
I: With the Interaction Term
WI: Without the Interaction Term
FE: Feature Engineering
DTHD: Days to heading
DTMT: Days to maturity
GL: Grain length
GW: Grain width
GH: Grain hardness
TKW: Thousand-kernel weight
TW: Test weight
SDS: Sodium dodecyl sulphate sedimentation
GP: Grain protein (GP)
PH: Plant height
